# Neuromedin U induces an invasive phenotype in CRC cells expressing the NMUR2 receptor

**DOI:** 10.1186/s13046-021-02073-8

**Published:** 2021-09-07

**Authors:** Patrycja Przygodzka, Ewelina Sochacka, Kamila Soboska, Marcin Pacholczyk, Izabela Papiewska-Pająk, Tomasz Przygodzki, Przemysław Płociński, Steven Ballet, An De Prins, Joanna Boncela

**Affiliations:** 1 Institute of Medical Biology, Polish Academy of Sciences, Lodowa 106, 93-232 Lodz, Poland; 2 Faculty of Biology and Environmental Protection, University of Lodz, Pomorska 141/143, 90-236 Lodz, Poland; 3 Department of Systems Biology and Engineering, Silesian University of Technology, Akademicka 16, 44-100 Gliwice, Poland; 4 Department of Haemostasis and Haemostatic Disorders, Chair of Biomedical Sciences, Medical University of Lodz, Mazowiecka 6/8, 92-235 Lodz, Poland; 5 Department of Immunology and Infectious Biology, Faculty of Biology and Environmental Protection, University of Lodz, Banacha 12/16, 90-237 Lodz, Poland; 6 Research Group of Organic Chemistry, Departments of Chemistry and Bioengineering Sciences, Vrije Universiteit Brussel, Pleinlaan 2, 1050 Brussels, Belgium

**Keywords:** Neuromedin U, NMUR2, GPCR, CRC migration and invasion

## Abstract

**Background:**

Successful colorectal cancer (CRC) therapy often depends on the accurate identification of primary tumours with invasive potential. There is still a lack of identified pathological factors associated with disease recurrence that could help in making treatment decisions. Neuromedin U (NMU) is a secretory neuropeptide that was first isolated from the porcine spinal cord, and it has emerged as a novel factor involved in the tumorigenesis and/or metastasis of many types of cancers. Previously associated with processes leading to CRC cell invasiveness, NMU has the potential to be a marker of poor outcome, but it has not been extensively studied in CRC.

**Methods:**

Data from The Cancer Genome Atlas (TCGA) were used to analyse *NMU* and NMU receptor (*NMUR1* and *NMUR2*) expression in CRC tissues vs. normal tissues, and real-time PCR was used for *NMU* and NMU receptor expression analysis. NMU protein detection was performed by immunoblotting. Secreted NMU was immunoprecipitated from cell culture-conditioned media and analysed by immunoblotting and protein sequencing. DNA demethylation by 5-aza-CdR was used to analyse the regulation of *NMUR1* and *NMUR2* expression. NMU receptor activity was monitored by detecting calcium mobilisation in cells loaded with fluo-4, and ERK1/2 kinase activation was detected after treatment with NMU or receptor agonist. Cell migration and invasion were investigated using membrane filters. Integrin expression was evaluated by flow cytometry.

**Results:**

The obtained data revealed elevated expression of *NMU* and *NMUR2* in CRC tissue samples and variable expression in the analysed CRC cell lines. We have shown, for the first time, that NMUR2 activation induces signalling in CRC cells and that NMU increases the motility and invasiveness of *NMUR2*-positive CRC cells and increases prometastatic integrin receptor subunit expression.

**Conclusions:**

Our results show the ability of CRC cells to respond to NMU via activation of the NMUR2 receptor, which ultimately leads to a shift in the CRC phenotype towards a more invasive phenotype.

**Supplementary Information:**

The online version contains supplementary material available at 10.1186/s13046-021-02073-8.

## Background

Colorectal cancer (CRC) is the third most common cancer worldwide, with 1.52 million new cases predicted in 2030 [1]. CRC is a highly heterogeneous disease, and local and/or distant recurrences are two critical processes that influence the survival rate of CRC patients [2]. Despite many attempts [3, 4], it is still necessary to identify pathological factors associated with disease recurrence and poor outcome that may lead to more explicit clinical staging and help in making treatment decisions. Our previous studies of CRC cell plasticity initiated by Snail transcription factor expression suggested that the small, secreted peptide neuromedin U (NMU) is actively engaged in early processes that lead to the invasiveness of CRC cells [5].

NMU was recently associated with several cancer types [6]. NMU was associated with cancer cell invasiveness and chemoresistance acquisition and proposed to be a potential diagnostic and/or prognostic marker in lung, breast, renal, endometrial and hepatocellular cancer [6, 7]. However, to date, the role of neuromedin U in CRC has not been extensively explored.

In this study, we report, for the first time, the role of the NMU peptide in CRC progression.

Physiologically, NMU has been predominantly found in the central nervous system and in the periphery. In the gastrointestinal tract, locally administered NMU leads to smooth muscle contraction, regulating intestinal motility and regional blood flow [8], but epithelial cells are not the source of the peptide in the gastrointestinal tract under normal conditions [9]. Human neuromedin U synthesis is tightly regulated at the genetic, epigenetic, transcriptional and post-transcriptional levels [6], regulating access to cellular microenvironments. Peptide precursors can be produced from three different transcripts, resulting in 147-, 158- or, the most abundant, 174-amino acid-long precursors [10] that include an *N*-terminal signal peptide for secretion [9]. Pre–pro-NMU cleavage generates NMU-25, whose bioactivity depends on a highly conserved, amidated pentapeptide at the C-terminus (-Phe-Arg-Pro-Arg-Asn-NH_2_) [11]. NMU peptide stability in plasma was reported to be low, with an estimated half-life of generally less than 5 min [12, 13], but it remains unknown which proteases degrade the peptides. As the poor pharmacokinetic profile of native peptides caused by proteolytic degradation was reported [14], a broad range of more stable analogues was generated. NMU analogues have been divided into two groups depending on their lead molecule, namely, full-length NMU or truncated NMU orthologues, such as NMU-8, all of which include amidated pentapeptide at the C-terminus [12].

All currently known NMU receptors (reviewed here [6]), including the main receptors (i.e., NMUR1 and NMUR2) and the alternative receptors (i.e., NTSR1/GHSR1b heterodimers and NMUR2S), belong to the superfamily of G protein-coupled receptors (GPCRs). These receptors are transmembrane receptors with GPCR structures, except for NMUR2S, which has been suggested to be a negative regulator of NMU signalling [15]. Both NMUR1 and NMUR2 have high homology, and according to previous reports, they both activate the same signalling pathways that involve inositol phosphates and calcium as secondary messengers [6].

Although neuromedin U was identified more than 30 years ago [16], there are still many questions regarding peptide processing, generation, secretion, activity and target cells; additionally, expression of the NMU receptors and responsiveness of cancer and other cells in the tumour microenvironment are not fully understood.

As NMU has not previously been studied in CRC, we first analysed publicly available patient datasets collected in The Cancer Genome Atlas (TCGA; http://tcga-data.nci.nih.gov/tcga/) to determine the expression of *NMU* and its receptor. Next, the tissue data were validated using a panel of molecularly heterogeneous CRC cell lines. Finally, we showed that NMU induced an invasive phenotype in *NMUR2*-positive cancer cells.

## Materials and methods

### Peptides

NMU-9 (Phoenix Pharmaceuticals, Burlingame, CA, USA) with comparable to NMU-25 affinity to human NMU receptors [9] with an exact, determined by us, half-life in human plasma t_1/2_ = 4.60 ± 0.4 min. NMURs agonists, which are NMU-8 analogues, were synthesized with a HPLC purity > 98 %, as described in literature [17]. The NMUR1 agonist SBL-NMU-21 (compound 28): t_1/2_ = 253.5 min, EC50 = 7.7 nM (value was calculated based on the IP3 pathway activation data), IC50 = 0.36 nM (value was calculated based on the competitive binding assay). NMUR2 agonist SBL-NMU-17 (compound 39): t_1/2_ = 3.37 ± 0.5 min, EC50 = 45.5 nM, IC50 = 13 nM [17]. All peptides were used as solutions in water. Peptide sequences and stability data are available in the Table S1 (Additional File 1).

### TCGA patient data set

We downloaded gene expression data (RNA Seq v2) as RSEM normalized logarithms of raw counts from TCGA colorectal adenocarcinoma dataset (TCGA_coad) using FirebrowseR package in R. We analysed 571 tumour samples and 50 tumour samples with corresponding normal tissue specimens. The data were further divided into different groups according to selected clinical attributes: tumour stage, tumour site, metastasis indicator, etc. Overall survival (OS) was measured from surgery until death and was censored for patients alive at the last follow-up. The survival data were analysed using the univariate log-rank tests available in Survival R package. To distinguish low and high expressing groups according to particular gene, we used the median to divide the ordered data set into two-halves, below median (low expression group) and above median (high expression group).

### Cell culture

Normal human colonic epithelial cells (CCD 841 CoN) and human colon carcinoma cell lines (Caco-2, SW480, SW620, HCT15, HCT116, and HT29) were obtained from the American Type Culture Collection (ATCC, Manassas, VA, USA). HEK293 R2_HA cell line overexpressing NMUR2 was kindly provided by prof. Gary B. Willars (University of Leicester). CCD 841 CoN and Caco-2 were cultured in MEM (Thermo Fisher Scientific, Waltham, MA, USA) supplemented with 10 or 20 % fetal bovine serum (FBS) respectively, 1 mM MEM Non-Essential Amino Acids (Thermo Fisher Scientific) and 1 mM sodium pyruvate (Sigma-Aldrich, MO, USA). SW480, SW620, HCT15 cells were grown in RPMI 1640 (with ATCC modification, Thermo Fisher Scientific), HCT116 and HT29 in McCoy 5A (Thermo Fisher Scientific), HEK293 R2_HA in high glucose DMEM, all supplemented with 10 % FBS (for HCT15 and HCT116 cells FBS was heat inactivated). All media were supplemented with penicillin / streptomycin (Thermo Fisher Scientific) and primocin (Invivogen, CA, USA). Cells were cultured at 37 ˚C in a 5 % CO_2_/95 % humidified air. Cells were routinely tested for mycoplasma (PlasmoTest; InvivoGen). Routine authentication of the cell lines was performed (Eurofins, Luxemburg) (Additional File 6).

### NMU cloning and stable clones generation

DNA coding NMU was obtained by reverse transcription of total RNA isolated from SW480 cells, by the use of SuperScript One-Step RT-PCR System with Platinum Taq DNA Polymerase (Thermo Fisher Scientific) with primers: 5’-AGCTAAGCTTGCCGAGATGCTGCGAACAGAGAG-3’ and 5’-GGTCAGCAGGGTTCATTTAACGCGGATCCAATAGC-3’. PCR product was cloned into pJET1.2/blunt with the Clone JET PCR Cloning Kit (Thermo Fisher Scientific) using HindIII and BamHI restriction sites and subsequently to the pcDNA 3.1(+) (Thermo Fisher Scientific). The obtained pcDNA-NMU was propagated in *Escherichia coli* TOP10, purified with GeneJET Endo-Free Plasmid Maxiprep Kit (Thermo Fisher Scientific), and sequenced. Plasmids containing the *NMU* sequence were transfected into HT29 cells using Amaxa Cell Line Nucleofector Kit R and Amaxa 4D nucleofector X Unit (Basel, Switzerland). Caco-2 was chemically transfected using the Xfect ™ RNA Transfection Reagent (Takara Bio Inc., Kusatsu, Japan). Subsequently, the cells were cultured in medium supplemented with Hygromycin B (Thermo Fisher Scientific) 400 µg/ml for HT29 and 250 µg/ml for Caco-2. The selection medium was refreshed every 48 h. After 4 weeks in culture, well-separated colonies were isolated. NMU expression was verified through Western blot analysis.

### mRNA isolation and real-time PCR analysis

Total RNA was isolated with the ReliaPrep™ RNA Cell Miniprep System (Promega, Madison, WI, USA). The quality control of isolated RNA was performed using the 2100 Bioanalyser (Agilent Technologies, Palo Alto, CA, USA) according to the manufacturer’s instructions. 1 µg of the isolated total RNA (RIN ≥ 8) was reverse transcribed using the High Capacity cDNA Reverse Transcription Kit (Applied Biosystems, CA, USA) according to the manufacturer’s instructions. Real time PCR for human *NMU, NMUR1, NMUR2, NTSR1, GHSR1b, GAPDH* and *β-actin* was performed using FastStart Essential DNA Probes Master or FastStart Essential DNA Green Master (Roche, Basel, Switzerland). TaqMan Gene expression probes and primers used in the present study are shown in Table S2 (Additional File 1). Amplification was performed on a Roche LightCycler 96. *GADPH* and/or *β-actin* mRNA transcripts were used as internal control genes. The amount of target in the various samples was calculated using the 2^−ΔCt^ relative quantification method with DataAssist v.3.01.

### 5-aza-CdR Treatment for NMUR1 and NMUR2 analysis

Cells were seeded on 6-well plates (Corning, NY, USA). After cells attachment (6 h), DNA methyltransferase inhibitor, 5-aza-2’-deoxycytidine (5-aza-CdR) (Sigma-Aldrich) was added at final concentration of 50 µM. Medium containing 5-aza-CdR was refreshed every 24 h. After 96 h, total RNA was isolated and analysed. The real-time PCR products were analysed on a 2 % agarose gel with ethidium bromide (Sigma-Aldrich) and visualized.

### Western blot

CRC cells were lysed with RIPA buffer (100 mM TRIS-HCl, pH 7.5, 300 mM NaCl, 0.2 % SDS with 1 % IGEPAL CA-630 and the Halt™ Protease Inhibitor Cocktail (Thermo Fisher Scientific)) with subsequent centrifugation (18,000 x g, 4 °C, 20 min). Total protein concentration in lysate was quantified by BCA method (Pierce BCA Protein Assay; Thermo Fisher Scientific). Samples were loaded and separated by SDS-PAGE (Mini-PROTEAN TGX Stain-Free Gels; Bio-Rad Laboratories, CA, USA) and transferred onto nitrocellulose membranes (Bio-Rad Laboratories). NMU protein was detected with rabbit anti-NMU antibody (Genetex, CA, USA), for ERK1/2 kinases analysis mouse anti-ERK1/2 (Santa Cruz Biotechnology, USA) and rabbit anti-pERK1/2 antibodies (Invitrogen) were used. In EVs (extracellular vesicles) purity analysis we used rabbit antibodies anti-GM130, anti-annexin V and anti-flotilin-1 (Cell Signaling Technologies, Danvers, MA, USA). Proteins used as loading controls were detected by mouse anti-β-actin, mouse anti-α-tubulin or rabbit anti-GAPDH antibody (Abcam, Cambridge, GB). We used secondary HRP-conjugated goat anti-mouse (Santa Cruz Biotechnology) or anti-rabbit antibodies (Invitrogen). The signal was detected by chemiluminescence (Thermo Fisher Scientific) with Kodak BioMax Light Film from Eastman Kodak (NY, USA).

### NMU immunoprecipitation and mass spectrometry analysis of the products

Cells were cultured on 75 cm^2^ flasks to 80–90 % confluency, next washed (PBS) and the medium was changed to 5 ml of fresh growth medium without FBS. After 48 h, 2 ml of conditioned medium (CM) were collected, centrifuged (1000 x g, 4 °C, 20 min) and immediately used for NMU immunoprecipitation performed with Dynabeads™ Protein A Immunoprecipitation Kit (Thermo Fisher Scientific) according to manufacturer’s instruction. 20 µl of Dynabeads were conjugated with 2 µg of rabbit anti-NMU antibodies (Abbexa, Cambridge, GB) or IgG antibodies from rabbit serum (Sigma-Aldrich) as a control. Antibodies-antigen complexes were eluted with SDS sample buffer with β-mercaptoethanol at 95 °C for 5 min and analysed by immunoblotting.

A part of the gel with HCT116 CM immunoprecipitation was fixed in methanol. The excised gel slice, corresponding to the NMU signal on the Western blot was send for the mass spectrometry identification (described in Additional File 1; commercial service at the Institute of Biochemistry and Biophysics of the Polish Academy of Sciences, Warsaw, Poland).

### Extracellular vesicles (EVs) isolation

HCT116 were washed with serum-free medium to remove any vesicles present in FBS of growth medium and then conditioned for 48 h in medium supplemented with 10 % exo-free FBS (previously ultracentrifuged as described here [18]). The medium was collected and sequentially centrifuged at 300 x g for 10 min and at 2,000 x g for 20 min. The supernatant was ultracentrifuged for 1.5 h at 100,000 x g using OPTIMA L-80 Ultracentrifuge and Type 45 Ti Rotor, Fixed Angle (Beckman Coulter, Inc). The pellets were washed with PBS and centrifuged for 1.5 h at 100,000 x g. All centrifugations were performed in 4 ºC. EVs pellets were lysed with RIPA buffer (composition as in Western blot) and analysed by immunoblotting with rabbit antibody anti-NMU and also with antibodies anti-flotilin 1, anti-annexin V (EVs markers) and anti-GM130 (Golgi membrane marker) for EVs characterisation and purity analysis.

### Ca^2+^ mobilization assay upon NMU treatment

Cells grown on poly-lysine-coated 48-well plates (Corning) for 24 h were stained for 15 min at 37 °C with Hoechst 33342 (Invitrogen). PBS rinsed cells were loaded with Fluo-4 AM (5 µM; Invitrogen) for 45–60 min at 37 °C in the presence of 0.5 % of pluronic-127 in the incubation medium (growth medium without FBS and PS, supplemented with 0.1 % BSA). Cells were rinsed with warm PBS and bathed in a final volume of 300 µl of incubation medium. Images were acquired using AxioVert fluorescence inverted microscope (Zeiss, Germany) with the use of 10 x dry objective (NA 0.3). First, an image of Hoechst 33342-stained nuclei was acquired. Next, a time-lapse image sequence in green channel (Fluo-4) was recorded with exposure time of 500 ms in the same field of view. After approximately 20 s of acquisition peptide was added and acquisition was performed for further 60 s. We tested the range of peptide concentrations, for NMU-9 (0.3, 0.7, 1 and 3 µM), NMUR1 agonist (30, 40, 60, 120 nM) and NMUR2 agonist (150, 200, 300 nM). For analysis of CRC cells we chose minimal concertation that induce significant fluorescence change. NMU-9 (1 µM) and NMUR2 agonist (200 nM ~ 5 times the EC50 determined in literature [17]). In case of NMUR1 agonist we chose for analysis 40 nM (~ 5 times of EC50 shown in literature [17]) as only single-cell reaction was observed with either concentration. Quantification of the effects is described in Additional Files 1 and 3. Representative movies description and links are available in Additional File 1.

### ERK1/2 activation analysis

Cells were grown to confluence 70–80 % into 6 well plates and incubated in serum-free media for 24 h prior to experiment. Subsequently, cells were treated with NMUR2 agonist SBL-NMU-17 (250 nM) or phorbol 12-myristate 13-acetate (PMA; 3 ng/ml) (InvivoGen) for indicated times. Cells were lysed with RIPA buffer (100 mM TRIS-HCl, pH 7.5, 300 mM NaCl, 0.2 % SDS with 1 % IGEPAL CA-630 and the Halt Protease and Phosphatase Inhibitor Cocktail (Thermo Fisher Scientific) with subsequent centrifugation (18,000 x g, 4 °C, 20 min). 10 µg of total protein was loaded and separated by 10 % SDS-PAGE and analysed by immunoblot. For kinase activation inhibition, cells were preincubated for 1 h with PD98059 inhibitor (20 µM) (Santa Cruz Biotechnology).

### Colony formation assay

Cells were seeded into 6 well plates at a density of 500 cells / well. After 7 days, formed colonies were fixed in cold methanol and acetic acid (3:1) and stained with hematoxylin and 1 % eosin (both from Chempur, Poland). Colonies (with more than 50 cells) were counted under the Nikon Eclipse TE 2000-U microscope (Nikon, Tokio, Japan) by two independent investigators.

### Cell migration and invasion assay

Migration and invasion assays were performed using an 8 μm pore size Transwell system (Thermo Fischer Scientific). First, 2 × 10^5^ cells, were incubated with CellTracker™ Green CMFDA Dye (Invitrogen) for 30 min at 37 °C with following washing step with PBS. Cells resuspended in 200 ul of growth medium without FBS were added inside the insert fixed in the well with 600 ul medium with 10 % FBS. For invasion assays, the chamber was pre-coated with 1 mg/ml Matrigel (Corning). Cells were incubated for 3 h (migration) or 6 h (invasion) at 37 °C. Cells on the apical side of the chamber were gently scraped off using cotton swabs, filters were washed in PBS and pictures were taken under the microscope Nikon Eclipse TE 2000-U. Pictures analysis was done with the use of FIJI image processing package [19].

### Flow cytometry

Cells were detached by accutase treatment (Thermo Fisher Scientific), resuspended in PBS / 1 % BSA / 2 mM EDTA and stained with fluorophore-conjugated antibodies for 30 min at RT in the dark or unconjugated antibodies for 45 min at RT. Washing step with PBS with 2 mM EDTA was performed. When needed, cells were stained with corresponding secondary antibodies conjugated to AlexaFluor 488 (Thermo Fisher Scientific) for 30 min at RT in the dark with following washing step (PBS). Antibodies details are collected in the Table S3 (Additional File 1). FACS analysis was performed using FACS LSR II BD flow cytometer (Becton Dickinson, Franklin Lakes, NJ, USA) equipped with BD FACSDiva Software. The results were analysed by FlowJo ™ 10.7.1 software (BD).

### Statistical analysis

The Shapiro-Wilk test was used to confirm the Gaussian distributions of raw data. Data non-departing from normal distribution are presented as the mean with SD; otherwise, medians with interquartile ranges or median with min-to-max range are used. The Student’s t-test or t-test with the Welch’s correction was performed to test the differences between groups for normally distributed data. The Mann-Whitney U test was performed to test the differences between groups of data with non-normal distributions. In the case of multiple comparisons, the differences among normally distributed data were tested with ANOVA followed by Dunnet’s test and data departing from normal distribution were tested with the Kruskal-Wallis test followed by Dunn’s multiple comparisons test. In the case of relative comparisons to hypothetical value, the Wilcoxon signed-rank test or one-sample t-test were used according to the data distribution. Fisher’s Exact test was used for testing the significance of differences in probability of invasion occurrence between groups with high and low NMU expression level.

## Results

### *NMU* and *NMUR2* are overexpressed in CRC tissues

To examine the *NMU* expression pattern in CRC, we initially analysed the RNAseq data of colorectal carcinoma collected in TCGA database, which includes clinical information and gene expression data for 621 patients (Additional File 4). The numbers of patients with CRC in stages Tis (carcinoma in situ), T1, T2, T3 and T4 were 1, 20, 105, 421 and 70, respectively (for 4 patients, information regarding the stage was not provided). The median age of the patients was 68 (IQR 58–76) years, and 46.7 % were women. From a clinical standpoint, 72.8 % of patients had colon cancer, and 26.2 % had rectal cancer (for 6 patients, tissue information was not provided). Paired data were analysed for 50 patients with gene expression data in cancer and normal adjacent tissues (Additional File 5).

**Fig. 1:**
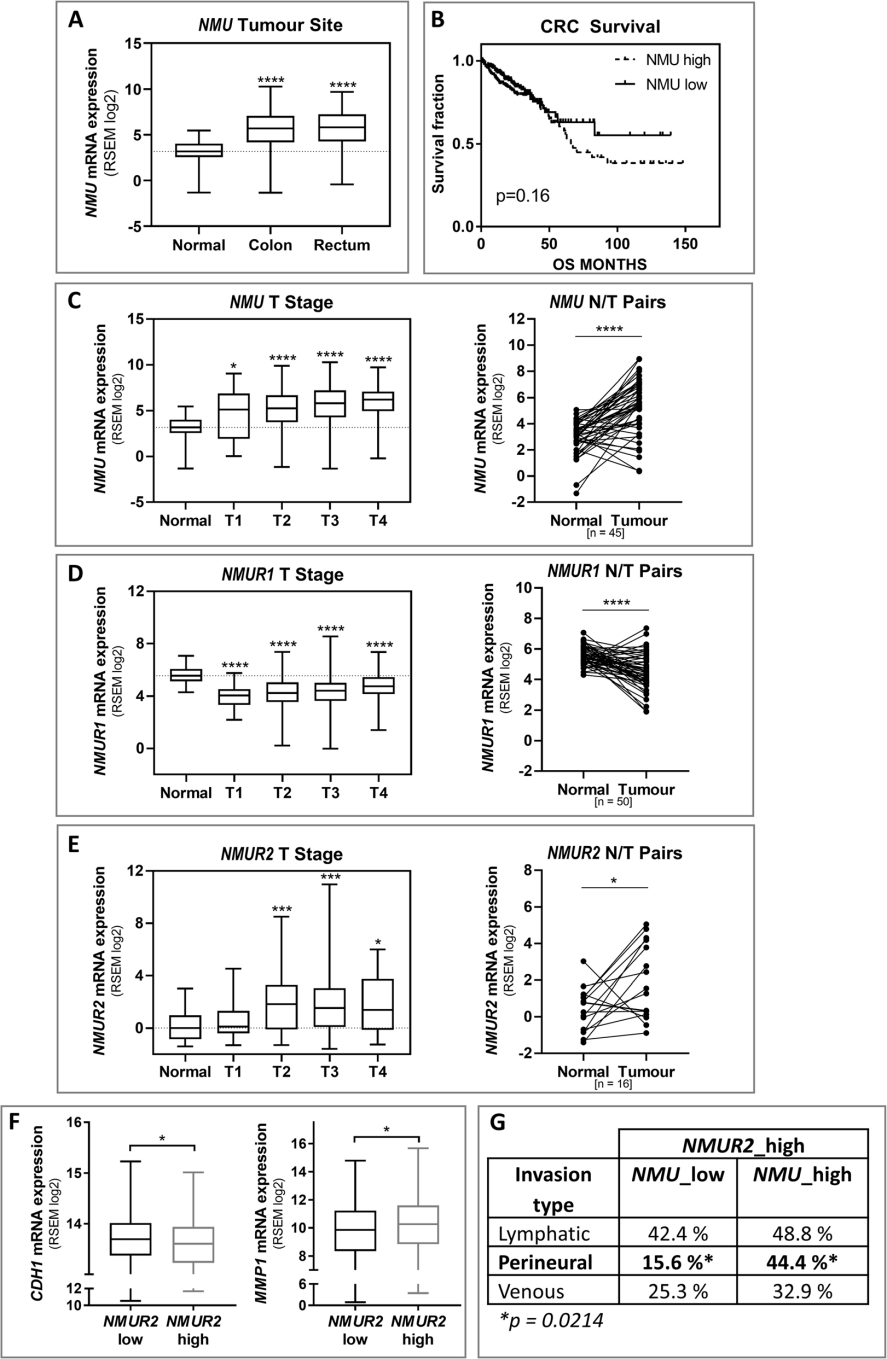
Human colorectal cancer tissues - TCGA data analysis. **A** The *NMU* expression values extracted from the TCGA RNA sequencing dataset were compared among CRC tumours of different sites and normal tissues. **B** Ordered patient data were divided into low *NMU* expression and high *NMU* expression groups according to the median expression level, and survival rates were compared between the groups by Kaplan-Meier analysis. **C** *NMU*, **D** *NMUR1* and **E** *NMUR2* expression extracted from the TCGA RNA sequencing dataset was compared among CRC tumours of different grades (T1-T4) and normal tissues. **F** *CDH1* and *MMP1* expression was analysed in the groups with low *NMUR2* and high *NMUR2* levels according to the median expression level. **G** The table shows the invasion types detected in the group with high *NMUR2* levels according to *NMU* mRNA abundance. For panels **A** and **C–F**, the boxes represent the interquartile range, the horizontal lines in the boxes represent the median, and the whiskers represent the minimum and maximum (*p ≤ 0.05, ***p ≤ 0.001, ****p ≤ 0.0001)

We observed a significant elevation in *NMU* expression in CRC tissue, regardless of tumour location and T stage, compared to normal tissue (Fig. 1A, C). Additionally, we confirmed elevated levels of *NMU* in tumour versus paired normal adjacent tissue samples (*n* = 45) (Fig. 1C). To assess the influence of the *NMU* expression level on CRC patient survival, we performed Kaplan-Meier analysis (Fig. 1B). Patients with high NMU levels had a tendency towards severe outcomes of lower overall survival, suggesting that NMU plays a role in CRC progression; however, this effect is not statistically significant.

Analysis of the mRNA expression of NMU receptors in the same CRC sample cohort revealed interesting changes. Although *NMUR1* was detected in both normal and tumour samples, its expression was significantly decreased in colorectal carcinoma at all stages (Fig. 1D), which was then confirmed in paired samples (*n* = 50). In contrast, *NMUR2* expression was found to be significantly increased in paired samples (*n* = 16) and tumours in the T2-T4 stages, but it was barely detectable in other samples (Fig. 1E). These findings show that *NMU* and *NMUR2* expression is upregulated in CRC tissue, which suggests the possible involvement of *NMU* in the disease process.

To characterize *NMUR2*-positive CRC samples, we analysed the level of *CDH1* (E-cadherin), a functional marker of cancer cell differentiation [20], and *MMP1*, a mediator of primary tumour invasion [21], in groups with various *NMUR2* expression levels. As shown in Fig. 1F, tissues with high *NMUR2* expression had significantly decreased *CDH1* and increased *MMP1* expression levels. Moreover, we observed significantly more perineural invasion (PNI), a prognostic factor of poor outcome, in samples with both high *NMUR2* and *NMU* expression (Fig. 1G). Taken together, these results suggest that high *NMUR2* and *NMU* expression correlates with decreased cancer differentiation and increased invasive potential.

### *NMU* expression in CRC cell lines of various phenotypes

To verify the observations from the above analysis of TCGA data, we chose six CRC cell lines representing distinct phenotypes typical of clinically defined CRC [3, 22, 23] (Table 1). The epithelial cell line CCD 841 CoN was used as the control normal epithelial cell line.

**Table 1:** CRC cell lines according to various phenotypes

Cell line	Disease/derived from	**CRC Subtype** by Schlicker et al. [22]	CMS Consensus Molecular Subtype [3]
**CCD 841 CoN**	Normal/colon epithelium	Normal epithelium	x
**Caco-2**	Colorectal carcinoma/primary tumour	1.2 Mesenchymal	**CMS4**
**HT29**	Colorectal adenocarcinoma/primary tumour	2.1 Epithelial	**CMS3**
**HCT15**	Colorectal adenocarcinoma/primary tumour	1.3 Mesenchymal	**CMS1**
**HCT116**	Colorectal carcinoma/primary tumour	1.2 Mesenchymal	**CMS4**
**SW480**	Colorectal adenocarcinoma/primary tumour	1.2 Mesenchymal	**CMS4**
**SW620**	Colorectal adenocarcinoma/lymph node metastasis	1.1 Strongly mesenchymal	**CMS4**

**Fig. 2:**
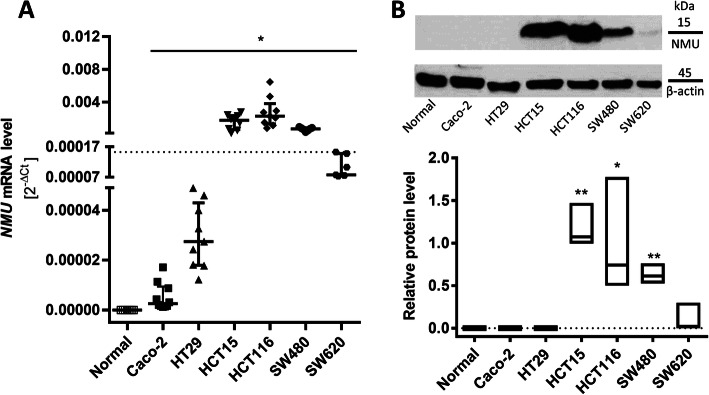
*NMU* expression in colorectal cancer cell lines. **A** *NMU* expression in the tested cell lines. The dotted line represents the median *NMU* expression in CRC cells (2^−ΔCt^ = 0.0001530). The results are shown as the median with interquartile range (*p ≤ 0.05; *n* ≥ 6). **B** NMU levels in cell lysates were analysed by immunoblotting. The image shows representative immunoblotting analysis results. The bands were quantified by densitometry. The intensity of the NMU band was normalized to that of the respective β-actin band (*p ≤ 0.05, **p ≤ 0.01; *n* ≥ 3). The results are shown as the medians with min-to-max ranges

All the tested CRC cells expressed *NMU*, but *NMU* expression was not detected in normal epithelial cells (Fig. 2A). This observation is consistent with our analysis of CRC patient sample data from TCGA. The median *NMU* expression in all the CRC cell lines was used to divide the cells into low *NMU* expression (Caco-2, HT29, and SW620 cells) and high *NMU* expression (HCT15, HCT116, and SW480 cells) groups. We observed that high peptide expression in the tested cell lines correlated with their mesenchymal phenotype and their primary tumour origin.

To determine the function of NMU in CRC, we analysed the NMU protein level. We confirmed the NMU protein levels in SW620 cell lysates and all cell lines with high NMU expression (HCT15, HCT116 and SW480 cells) (Fig. 2B). Nevertheless, the *NMU* mRNA analysis suggests that the possibility of NMU peptide production in other CRC cell lines cannot be excluded. The protein level of NMU may have been below the limit of detection of the method used.

### *NMU* secretion by CRC cells

As NMU is a secretory peptide, we determined its levels in CRC cell-conditioned media. We performed immunoprecipitation of NMU from CRC cell-conditioned media using an anti-NMU antibody (immunogen: AA 35–158) that recognizes both the longer pre-pro-peptide and the shorter NMU-25 peptide.

**Fig. 3:**
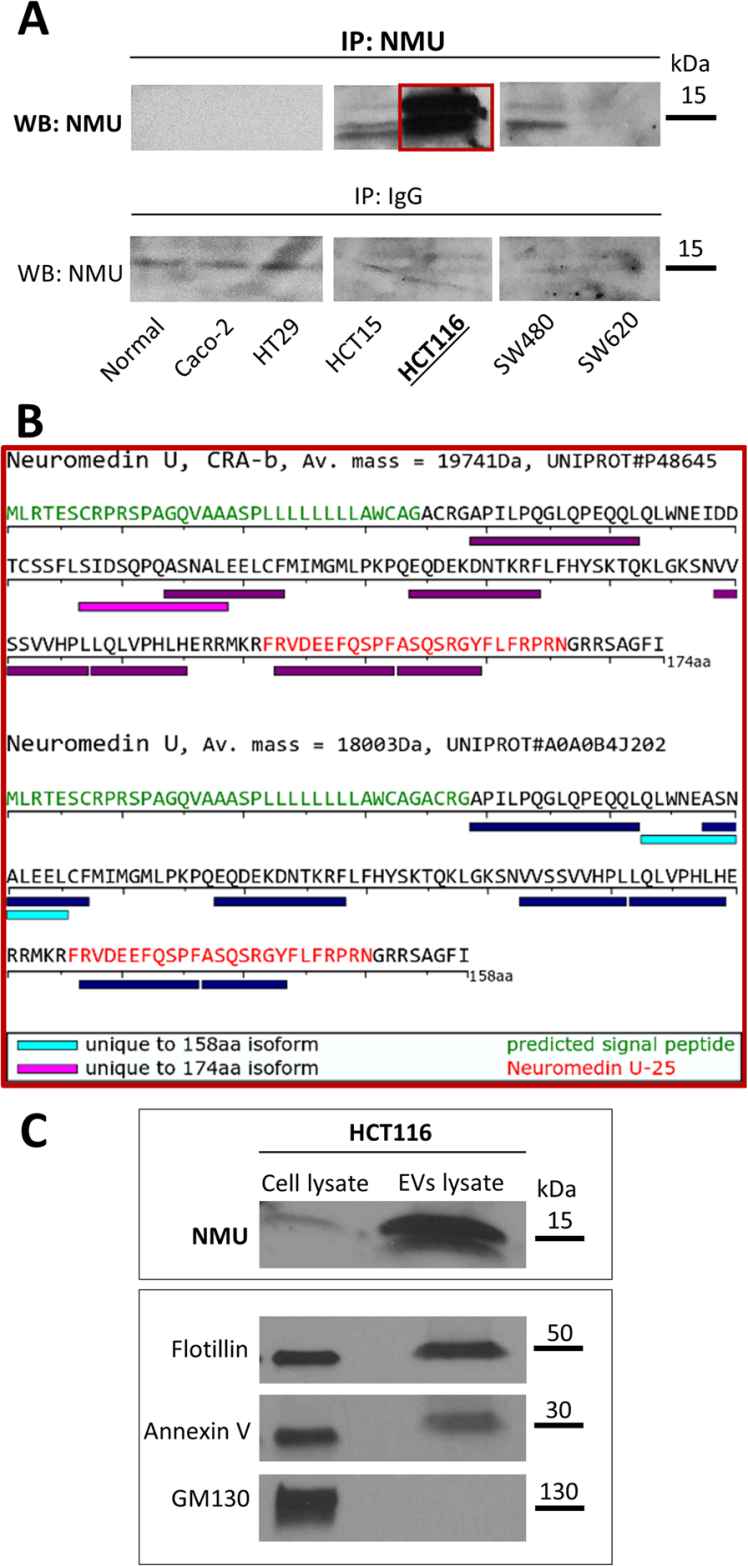
NMU secretion by colorectal cancer cells. **A**  Secreted NMU was immunoprecipitated from the conditioned media (CM) of CRC cells and analysed by immunoblotting. The image shows representative immunoprecipitation analysis results. **B** Immunoprecipitated proteins from the CM of HCT116 cells were excised from the gel and subjected to mass spectrometry analysis. PEAKS X Studio analysis of the resulting spectra indicated that two types of NMU isoforms were secreted based on the detection of unique peptides. **C **Extracellular vesicles released from HCT116 cells were isolated from the conditioned media and lysed, and the levels of NMU, EV markers and EV purity indicators were analysed by immunoblotting (*n* = 3)

By NMU immunoprecipitation, we detected the NMU protein in multiple forms of partially processed peptides in the conditioned media from all the cell lines with high NMU expression (HCT15, HCT116 and SW480 cells) (Fig. 3A). However, we cannot exclude the possibility that the sensitivity of the method was insufficient to detect NMU in the conditioned media from the cell lines expressing low levels of NMU.

Mass spectrometry analysis of the products immunoprecipitated from HCT116 cells, which exhibited the highest NMU expression, revealed the presence of unique peptides from the 174-AA and 158-AA precursor forms of NMU (Fig. 3B). This means that NMU is produced from two distinct transcripts, released and cleaved.

Subsequently, we searched for NMU in extracellular vesicles (EVs) isolated from the media of HCT116 cells to determine whether NMU is sorted to EVs by CRC cells, which occurs for many other secretory proteins [24]. Indeed, NMU was detected among the cargo of extracellular vesicles isolated from the HCT116 cell-conditioned media (Fig. 3C). This indicates that NMU is released as a mixture of processed peptide forms that can be loaded into vesicles, which enables distant transport without exposure to proteolytic cleavage.

### *NMUR1* and *NMUR2* receptor expression in CRC cell lines

**Fig. 4:**
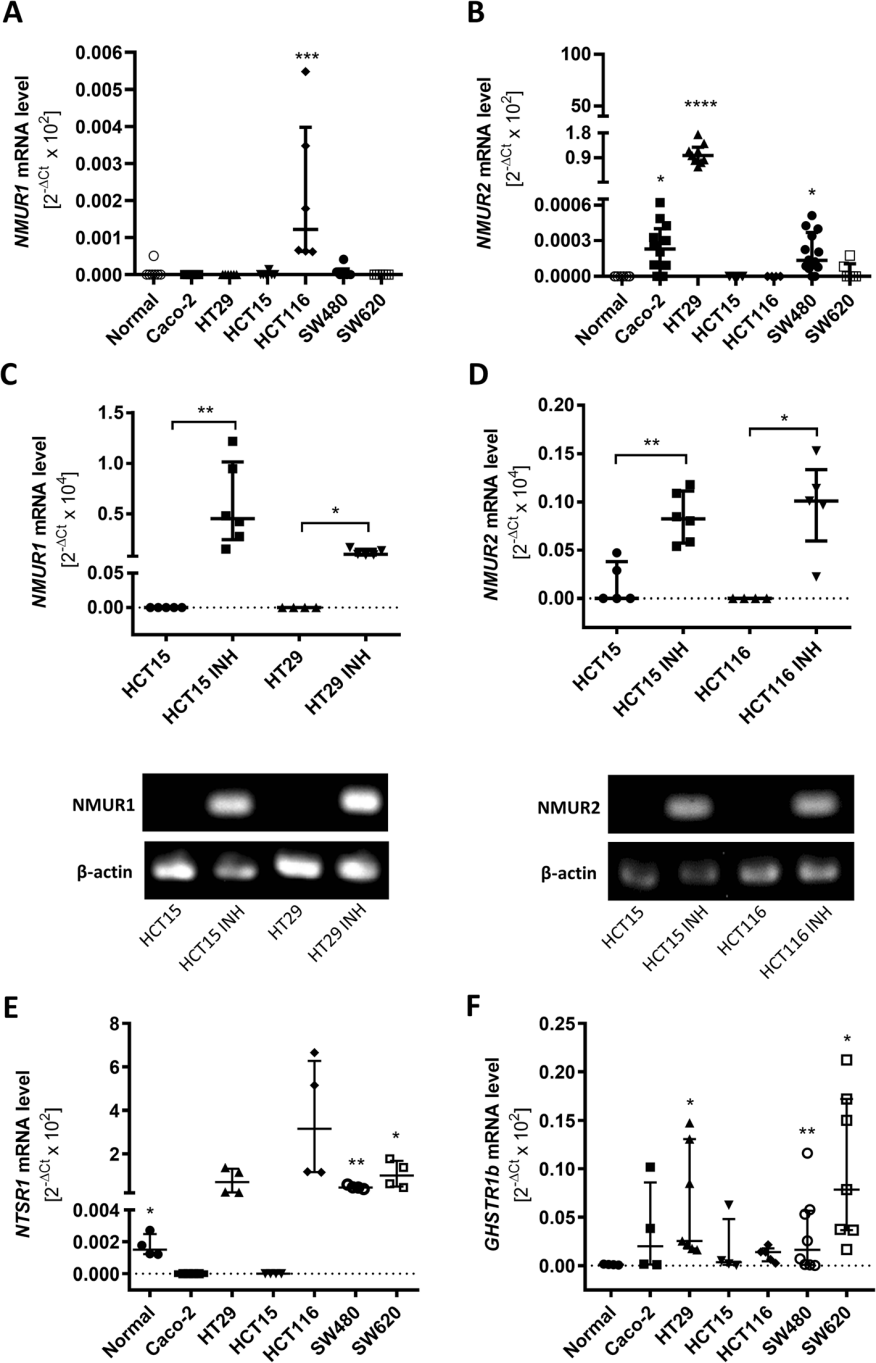
NMU receptor expression in normal epithelial and colorectal cancer cells. **A** *NMUR1* and **B** *NMUR2*  mRNA levels were analysed in cancer cells and compared to those in normal epithelial cells (*p ≤ 0.05, ***p ≤ 0.001, ****p ≤ 0.0001; *n* ≥ 6). **C** *NMUR1* and **D** *NMUR2* expression levels were analysed in cells treated with 50 µM 5-aza-CdR inhibitor (INH) for 4 days. *GAPDH* or *β-actin* was used as the internal control (*p ≤ 0.05, ***p ≤ 0.001; *n* ≥ 4). The results are shown as the medians with interquartile ranges. The images show representative results of electrophoretic analysis of real-time PCR products. **E** *NTSR1* and **F** *GHSR1b* mRNA levels were analysed in cancer cells and compared to those in normal epithelial cells. *GAPDH* or *β-actin* was used as the internal control. The results are shown as the median with interquartile range (*p ≤ 0.05, **p ≤ 0.01; *n* ≥ 4)

As CRC cell lines exhibit variable NMU expression, we assumed that these cell lines also differ with respect to their responsiveness to NMU. Since NMU performs its function by binding to specific receptors, we analysed the mRNA levels of *NMUR1* and *NMUR2*. HCT116 cells were the only cell type that expressed *NMUR1* (Fig. 4A). The highest expression of *NMUR2* was observed in HT29 cells, but *NMUR2* expression was also detected in Caco-2 and SW480 cells (Fig. 4B). Three out of the four cell lines with receptor expression (Caco-2, HCT116, and SW480 cells) belong to the CRC consensus molecular subtype CMS4, which is associated with poor outcome, and the remaining cell line (HT29 cells) belongs to the subtype CMS3, which includes epithelial tumours with dysregulated metabolism [3, 4]. Interestingly, no expression of NMU receptors was detected in HCT15 cells, although they exhibited high NMU secretion. These results suggest that some CRC cells secrete NMU but are likely unable to respond to NMU.

Because differences in NMUR expression were observed, we determined whether NMUR expression is regulated at the transcriptional level, as shown for neurotensin receptor 1 (NTSR1), a receptor subtype closely related to NMURs, in primary colorectal tumours [25]. Cell treatment with 5-aza-CdR showed that the expression of both receptors was indeed regulated by promoter methylation (Fig. 4C, D).

To complete the picture of NMU receptor expression by CRC cells, we analysed the expression of the alternative receptors *NTSR1/GHSR1b* [26] and *NMUR2S* [15], which is a truncated form of *NMUR2*, by real-time PCR. We demonstrated the gene expression of *NTSR1* and *GHSR1b* and observed that NTSR1/GHSR1b heterodimer formation is possible in SW480 and SW620 cells (Fig. 4E, F). Using previously published primer sequences [15], we were unable to detect the *NMUR2S* transcript in the CRC cell panel.

### Activity of NMU receptors in CRC cells

Previous studies concerning NMU signalling in cancer cells were performed on cell models with exogenously overexpressed NMU receptors. Here, we detected the activity of endogenous NMU receptors in CRC cells.

Initially, we attempted to detect Ca^2+^ mobilisation and the phosphorylation of extracellular signal-regulated kinases 1 and 2 (ERK1/2) in CRC cell lines treated with NMU-9. We were not able to detect statistically significant changes. Thus, we used NMU-8 analogues, i.e., an NMUR1 agonist (SBL-NMU-21) or NMUR2 agonist (SBL-NMU-17), that have defined affinities for NMURs. The NMUR1 agonist has a significantly higher half-life in human plasma than native NMU [17] and the NMUR2 agonist has similar stability to native NMU (Table S1, Additional File 1).

**Fig. 5:**
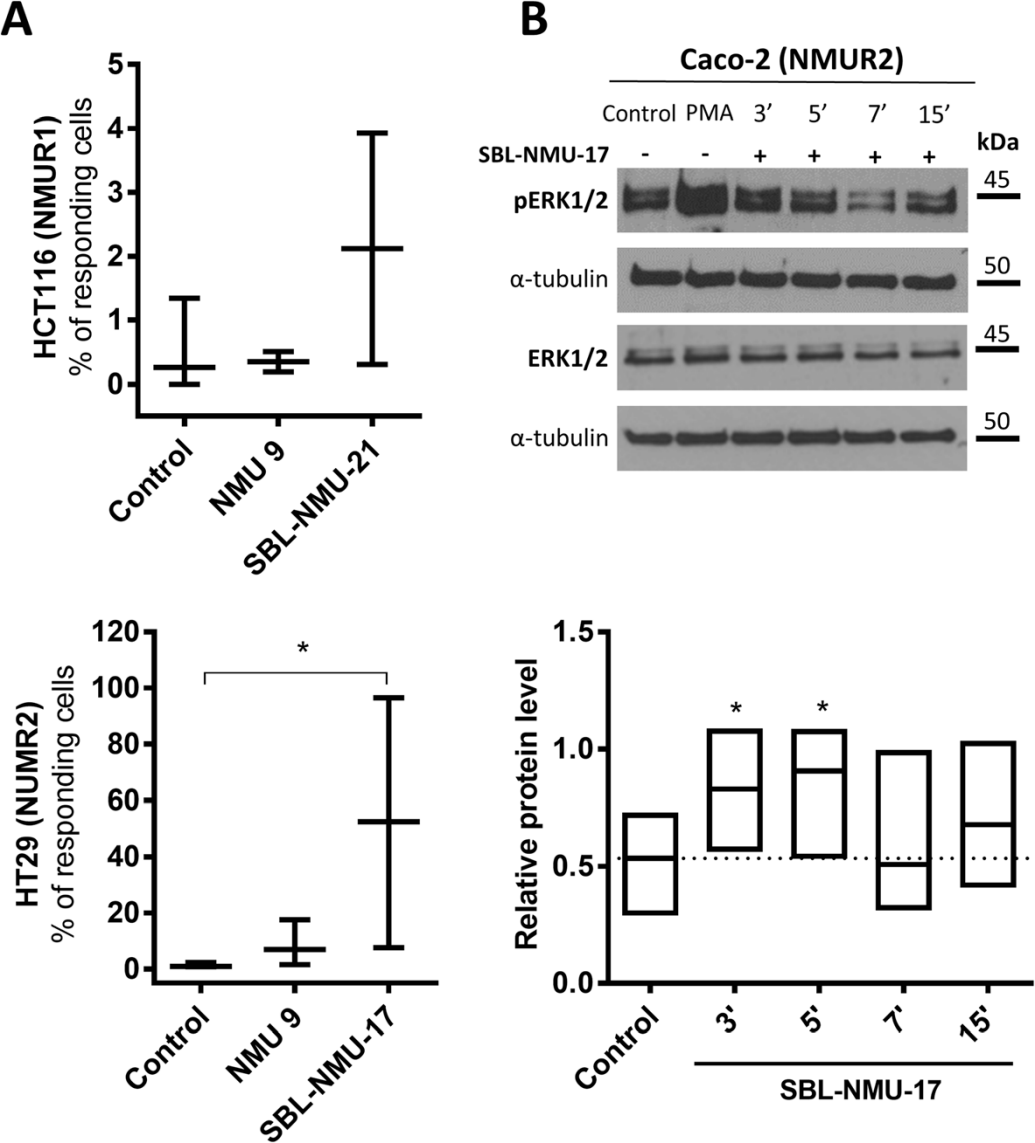
*NMU* receptor activity in colorectal cancer cells.** A **Ca^2+^  influx after NMU treatment of HCT116 (*upper panel*) and HT29 (*lower panel*) cells loaded with fluo-4. Cells were treated with NMU or an NMUR1 (SBL-NMU-21) or NMUR2 (SBL-NMU-17) agonist. The changes in fluorescence were detected and analysed as described in the Methods section. The results are shown as the mean with SD (*p ≤ 0.05; *n* = 5). **B** ERK1/2 kinase activation after SBL-NMU-17 NMUR2 agonist treatment of Caco-2 cells was analysed by immunoblotting. The image shows a representative result. The bands were quantified by densitometry. The intensity of the pERK1/2 bands was normalized to those of the respective ERK1/2 and α-tubulin bands. The results are shown as the median with min-to-max range (*p ≤ 0.05; *n* = 5)

To detect changes in the fluorescence of fluo-4 that result from Ca^2+^ mobilisation induced by NMU, we selected the cell lines with the highest expression of *NMUR1* (HCT116 cells) and *NMUR2* (HT29 cells). Next, we selected the minimal effective concentration of each compound for further analysis. We used the following concentrations: 1 µM NMU-9, 40 nM NMUR1 agonist and 200 nM NMUR2 agonist (Fig. 5A and representative movies, Additional File 1). We did not detect statistically significant changes in the fluorescence intensity caused by Ca^2+^ mobilisation in HCT116 cells treated with NMU-9 or the NMUR1 agonist (SBL-NMU-21) (Fig. 5A, *upper panel*), but HT29 cells significantly responded to treatment with the NMUR2 agonist (SBL-NMU-17) (Fig. 5A, *lower panel*). To show that the observed signal depends on *NMUR2* expression, we investigated the HEK293 R2_HA clone with high exogenous expression of *NMUR2* (Fig. S1 A, Additional File 2) and observed significant calcium mobilisation after treatment with both NMU-9 and the NMUR2 agonist (Fig. S1 B, Additional File 2).

The observed high variability in the response of CRC cells did not allow us to reliably estimate curve parameters, such as EC50, to compare the effects between the NMU and NMU analogues or between cell lines. We speculate that receptor activity is dependent on the cell culture density or local pericellular peptide concentration but it needs to be thoroughly explored in the future.

Subsequently, we analysed the phosphorylation of ERK1/2 kinases, as they are major cellular effectors of CRC progression and are activated by GPCRs [27]. We analysed ERK1/2 activation in the Caco-2 cell line (the only *BRAF* and *KRAS* wild-type cells in our CRC panel with an intact MAPK pathway [23]) after NMU-9 and NMUR2 agonist treatment. We did not detect significant phosphorylation of ERK1/2 induced by NMU-9 (Fig. S2, Additional File 2) but the SBL-NMU-17 NMUR2 agonist induced ERK1/2 kinase activation with the peak effect detected after 3 and 5 min of treatment (Fig. 5B), regardless of the relatively low *NMUR2* expression in Caco-2 cells. Again, to show that the observed signal depends on NMUR2, we investigated the HEK293 R2_HA clone and observed ERK1/2 phosphorylation as a result of treatment with both NMU-9 and the NMUR2 agonist (Fig. S1 C&D, Additional File 2).

### Morphology and proliferation of CRC cells characterized by *NMUR2* expression and elevated *NMU* production

**Fig. 6:**
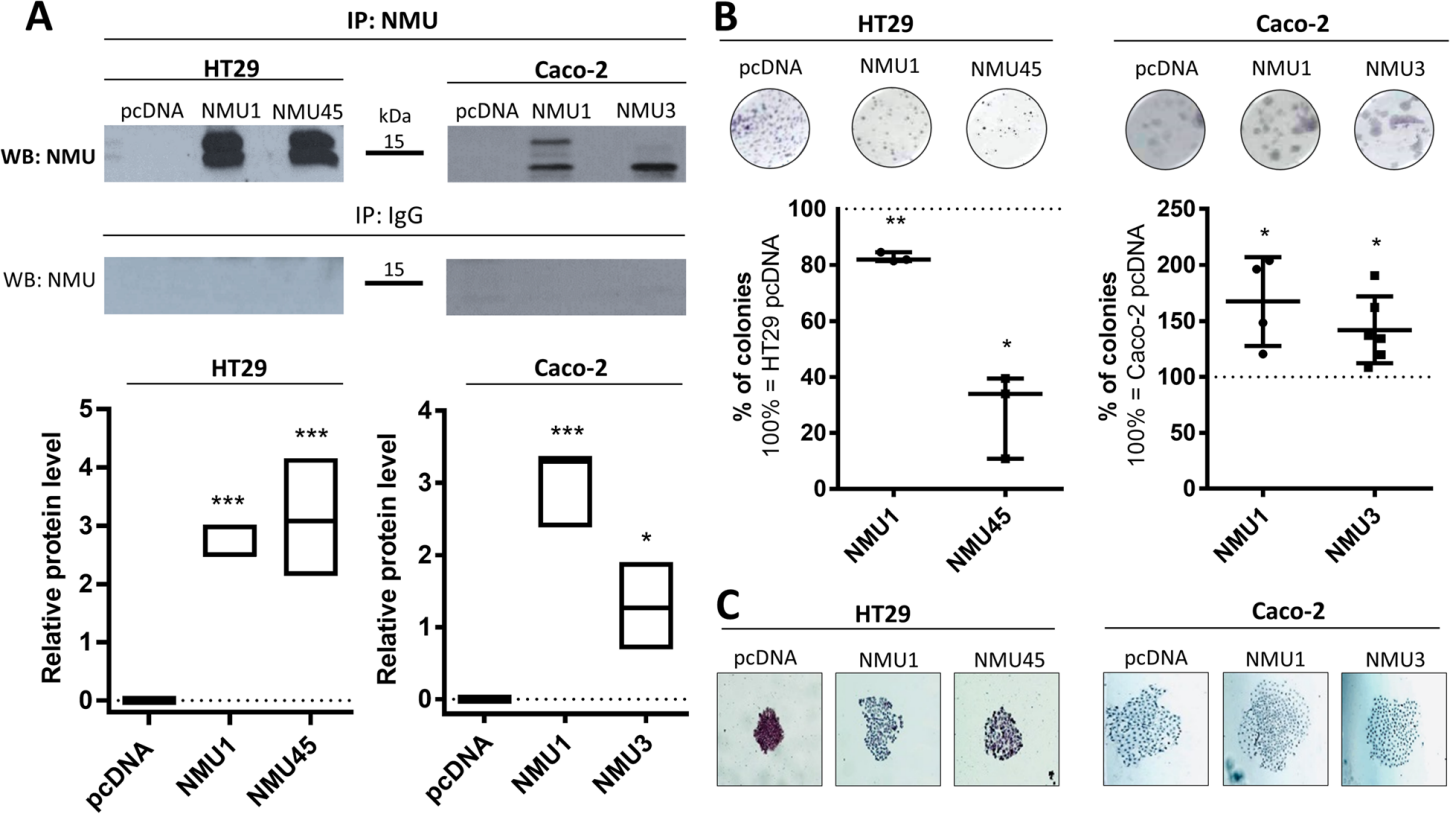
NMU secretion, colony formation and morphology of NMU-overexpressing HT29 and Caco-2 clones.** A **NMU secretion by HT29 (*left*) and Caco-2 (*right*) NMU-overexpressing clones was confirmed by NMU immunoprecipitation from the cell-conditioned media. The bands were quantified by densitometry. The results are shown as the median with min-to-max range (*p ≤ 0.05, ***p ≤ 0.001; *n* = 3). The images (*upper panel*) show representative results. **B** Clonogenic capabilities of the NMU-overexpressing clones. The images (*upper panel*) show representative colony densities. The results are shown as the median with interquartile range (*p ≤ 0.05, **p ≤ 0.01; *n* ≥ 3). **C** Morphology of the HT29 and Caco-2 NMU-overexpressing clone colonies. HT29 pcDNA and Caco-2 pcDNA – control clones transfected with an empty vector; HT29 NMU1, HT29 NMU45, Caco-2 NMU1, Caco-2 NMU3 – various clones overexpressing NMU

To analyse the role of NMU signalling in CRC cells, we generated NMU-overexpressing clones. We chose HT29 and Caco-2 cells with various levels of *NMUR2* expression and low expression of NMU since we observed an increase in *NMUR2* expression in CRC by TCGA data analysis and a response of *NMUR2*-positive cells to NMU. HT29 cells are a low invasive, metastatic epithelial cell line classified as CMS3 in contrast to highly invasive, nonmetastatic Caco-2 cells, which have a mesenchymal phenotype and CMS4 features. We generated two NMU-overexpressing clones per cell line, namely HT29 NMU1, HT29 NMU45, Caco-2 NMU1, and Caco-2 NMU3 and one control clone per cell line that was transfected with the empty vector pcDNA, namely HT29 pcDNA and Caco-2 pcDNA. NMU overexpression (Fig. S3; Additional File 2) and secretion (Fig. 6A) were confirmed by immunoblotting and NMU immunoprecipitation from the conditioned media.

We observed that NMU overexpression had different effects on the features of the studied cell lines. The HT29 NMU clones formed fewer colonies from a single cell than the control clone (Fig. 6B, *left*), and tight colonies became more dispersed with relaxed tight junctions (Fig. 6C, *left*). NMU overexpression did not affect the spread of the Caco-2 colonies (Fig. 6C, *right*), but the ability of the Caco-2 NMU clones to form colonies from a single cell was increased (Fig. 6B, *right*). The proliferation of the NMU clones of both lines was not affected (data not shown). These data show that NMU modifies the phenotypes of colorectal cancer cells regardless of their epithelial or mesenchymal phenotype.

### *NMU* expression and secretion accelerate *NMUR2*-positive CRC cell migration and invasion via ERK1/2 kinase activation

**Fig. 7:**
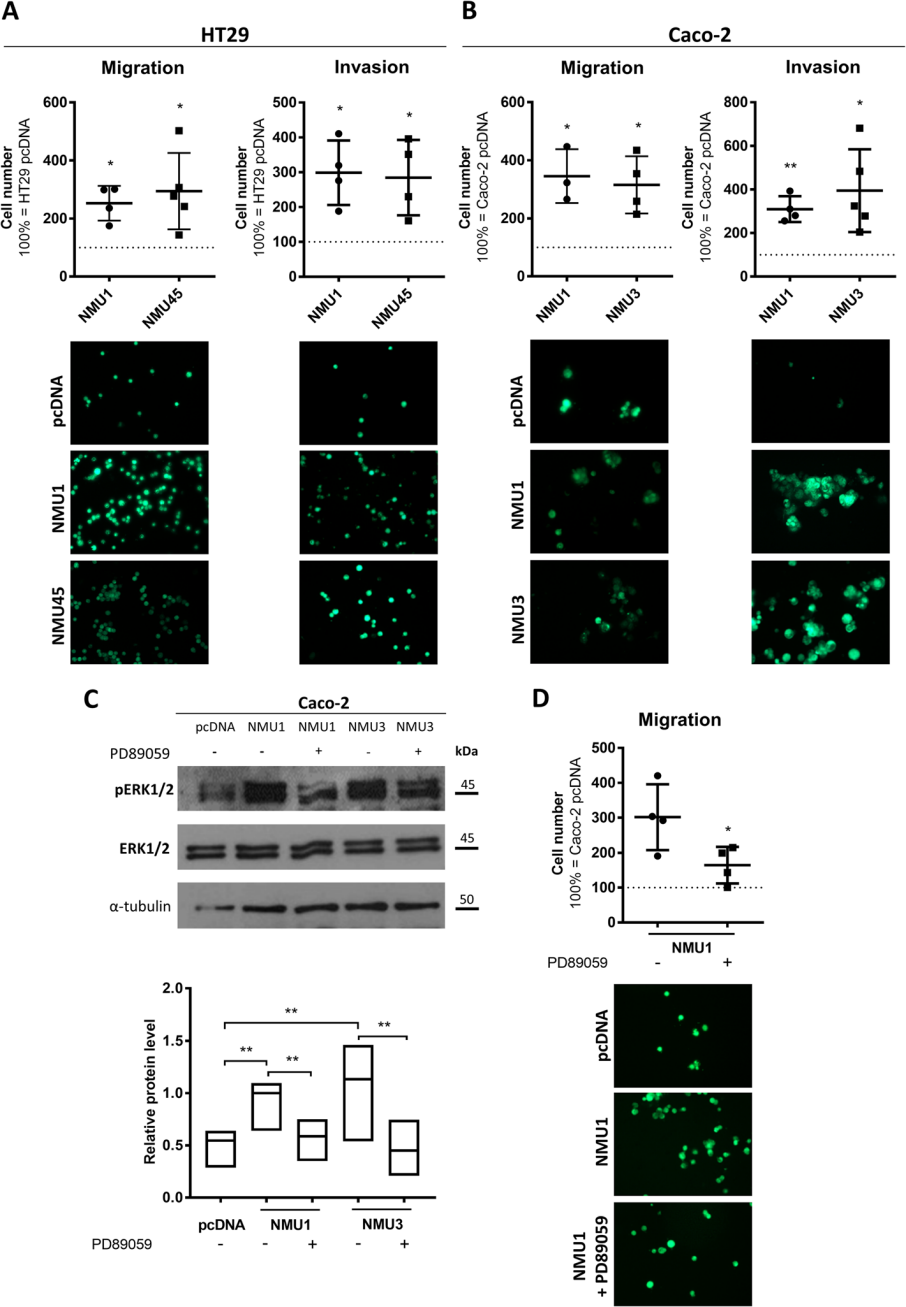
Migration and invasiveness of colorectal cancer cells overexpressing NMU. **A** HT29 NMU and **B** Caco-2 NMU clone migration (*left panel*) and invasion (*right panel*) capabilities compared with those of control pcDNA clones. The results are shown as the mean with SD (*p ≤ 0.05, **p ≤ 0.01; *n* ≥ 3). The images show representative results. **C **ERK1/2 kinase activation in the Caco-2 NMU clones treated with the ERK1/2 phosphorylation inhibitor PD89059 was analysed by immunoblotting. The image shows a representative result. The bands were quantified by densitometry. The intensity of the pERK1/2 bands was normalized against that of the respective ERK1/2 and α-tubulin bands. The results are shown as the median with min-to-max range (*p ≤ 0.05, **p ≤ 0.01; *n* ≥ 4). **D** Caco-2 NMU1 clone migration was inhibited by the PD89059 inhibitor. The results are shown as the mean with SD (*p ≤ 0.05; *n* = 4). The images show representative results. HT29 pcDNA and Caco-2 pcDNA – control clones transfected with an empty vector; HT29 NMU1 and HT29 NMU45, Caco-2 NMU1 and Caco-2 NMU3 – various clones overexpressing NMU

The observed changes in clone characteristics suggest that NMU can stimulate the invasive potential of CRC cells expressing *NMUR2*. To verify this hypothesis, we investigated the HT29 and Caco-2 NMU clones in migration and invasiveness assays. As shown in Fig. 7, all the clones that overexpressed NMU migrated faster (Fig. 7A, B) and had significantly increased invasive properties (Fig. 7A, B) regardless of the parental cell phenotype. Notably, even the mesenchymal Caco-2 cells became more invasive.

As NMU acts through NMUR2, we investigated the ERK1/2 kinase activity in the Caco-2 NMU clones. We observed higher activity of the ERK1/2 signalling pathway, which was sensitive to PD98059 (an ERK1/2 phosphorylation inhibitor), in both clones (Fig. 7C). Moreover, the migratory potential of the NMU clones depended on the ERK1/2 signalling pathway and was significantly reduced when the pathway was disrupted (Fig. 7D).

### *NMU* overexpression leads to metastatic/promigratory integrin overexpression

**Fig. 8:**
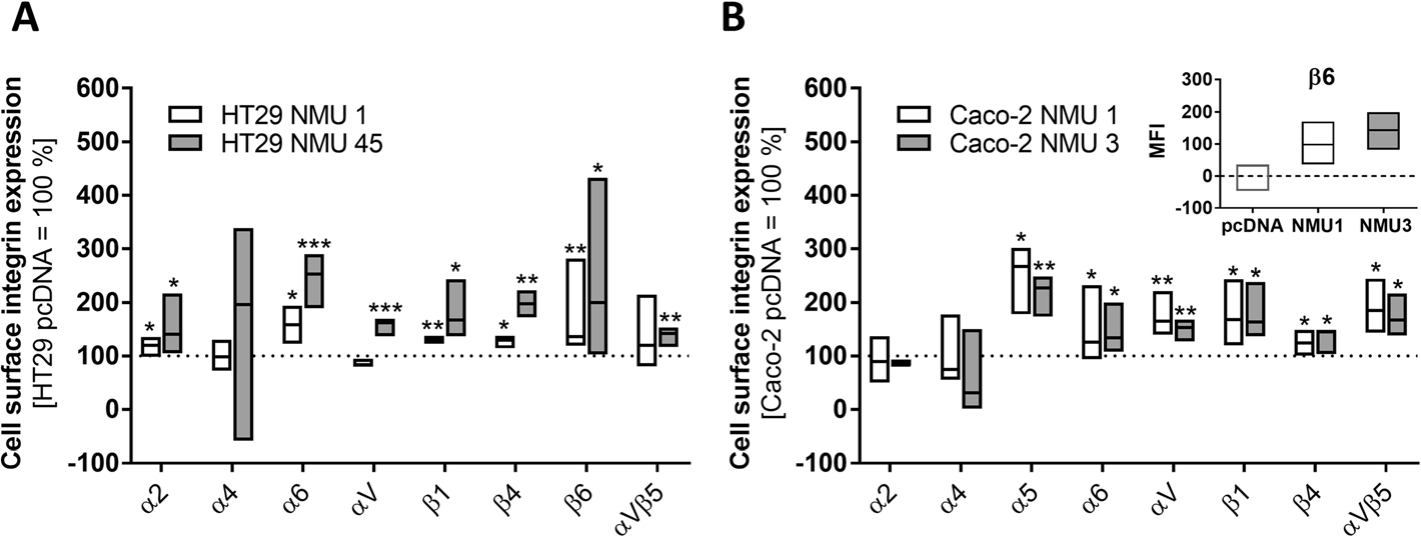
Cell surface integrin receptor subunit expression in NMU clones.The relative expression levels of cell surface integrins in **A** the HT29 NMU and **B** Caco-2 NMU clones were analysed by flow cytometry. The results show the relative fluorescence intensity in the NMU clones compared with that in the control clones (either HT29 or Caco-2 cells transfected with the empty vector pcDNA). The inset in panel **B** shows the median fluorescence intensity of the β6 subunit. The results are shown as the median with min-to-max range (*p ≤ 0.05, **p ≤ 0.01, ***p ≤ 0.001; *n* ≥ 3). HT29 pcDNA and Caco-2 pcDNA – control clones transfected with an empty vector; HT29 NMU1 and HT29 NMU45, Caco-2 NMU1 and Caco-2 NMU3 – various clones overexpressing NMU

Increases in the invasive properties and mobility of CRC cells are often associated with changes in integrin expression. Indeed, flow cytometry analysis showed that the HT29 NMU45 clone exhibited an increased expression of the αV subunit and αVβ5 integrin. Both the HT29 NMU clones expressed increased levels of the α2, α6, β1, β4 and β6 subunits on the cell surface (Fig. 8A). Similarly, NMU overexpression resulted in increased levels of the α5, α6, αV, β1, β4, and β6 integrin subunits and increased levels of αVβ5 integrin in the Caco-2 NMU clones (Fig. 8B).

## Discussion

There is a broad spectrum of NMU engagement in cancer, as recently reviewed by our group [6]. A substantial proportion of these findings suggests a critical contribution of NMU to cancer growth and supports the hypothesis that NMU is a marker of poor prognosis and short patient survival [7, 26, 28–31]. Other reports have correlated significant shifts in peptide expression under metastatic conditions with a possible contribution of NMU not only to cancer development and growth but also to cancer progression [26, 28–30, 32]. Here, we add new observations regarding NMU expression, secretion, signalling and action in CRC.

TCGA data analysis enabled us to report, for the first time, higher *NMU* and *NMUR2* expression in CRC tissues than in normal epithelium and, similar to other cancer types [6], a noticeable trend of lower overall survival of patients with high *NMU* expression in their cancer tissues. We attempted to search for the significance of the above observations and the underlying molecular mechanisms.

First, we demonstrated that CRC cells are the source of NMU and that NMU expression correlates with its secretion. Moreover, we showed that NMU was secreted in the form of partially proteolytically processed 174-AA and 158-AA pre-pro-peptides. Intriguingly, although there is a growing number of reports regarding NMU activity in cellular microenvironments, the process of its generation from longer precursors, the time and place of proteolytic processing, the location and efficiency the *C*-terminal amidation process remain unknown [33, 34]. We show that NMU is also released as a cargo of extracellular vesicles produced by CRC cells. EVs encapsulate multiple proteins and other biomolecules to transport them; therefore EVs play important roles in intercellular communication and may facilitate tumour angiogenesis and metastasis [35]. Our observation of the presence of NMU in CRC EVs is consistent with the reported abundance of NMU in breast cancer EVs [36] and suggests that NMU plays complex roles in processes involved in cancer progression.

Since NMU exerts its function by binding to receptors, we asked whether its action occurs in an autocrine manner and whether the cancer cells themselves are its targets. To answer this question, we analysed NMU receptor expression in CRC samples and cells. In our TCGA data analysis, it appeared that there was higher *NMUR2* expression and lower *NMUR1* expression in CRC tissues than in normal tissues. In the CRC cell panel, NMUR expression was variable, as also shown in cell lines derived from other cancers [30, 32]. We determined that HCT15 and SW620 cells have no or vestigial expression of NMURs, HCT116 cells express *NMUR1*, and Caco-2, HT29 and SW480 cells express *NMUR2*. NMU is able to activate signalling pathways in CRC cells expressing NMU receptor. In light of the observed diversity in receptor expression, we focused on the regulation of NMUR expression. Recent analysis of primary colorectal tumours, including adenomatous and malignant portion of tumours, revealed that neurotensin receptor 1 (NTSR1), which is closely related to NMURs, was frequently methylated in the adenomatous portion, while methylation levels were lower in the cancerous portion [25]. These data support the opinion that cancer-associated DNA hypomethylation is also critical in human carcinogenesis [37]. Indeed, our results show that both NMURs are likely regulated via DNA methylation. This raises the possibility that this process allows single cells to switch between NMUR1 and NMUR2 expression what determines cellular phenotype and NMU responsiveness. Unfortunately, we faced the main limitation in the field of NMU receptors, which is the lack of reliable antibodies that discriminate between human NMUR1 and NMUR2. Most previously published conclusions were drawn from transcript and activity studies of cells with high exogenous NMUR expression or mouse models.

To determine whether CRC cells respond to NMU, we also examined the level of alternative NMU receptors in our cell line panel. We concluded that the possibility of NTSR1/GHSR1b heterodimer formation and NMU signalling through this receptor cannot be excluded in SW480 and SW620 cells (Fig. 4E, F). By investigating the activity of NMURs, we successfully showed signalling activation in *NMUR2*- positive cells; however, in this case, only the NMUR2 agonist, not NMU-9, was able to significantly trigger the signal. As an NMUR2 agonist (SBL-NMU-17) has NMUR2 affinity and potency comparable to the shorter form of NMU [17] and no other receptor binding of unmodified NMU is expected, the proteolytic instability of NMU-9 in CRC cell culture could be a culprit of a weak calcium mobilisation signal or a lack of ERK1/2 signalling activation. It is known that CRC cells produce proteolytic enzymes at concentrations that linearly increase with cell invasiveness and tumour progression [38]. We suggest that the observed discrepancies between the actions of NMU-9 and NMUR2 agonist in our studies may result from low NMU peptide stability in CRC cell culture and from the altered chemical structure of SBL-NMU-17. In our plasma stability studies, both the NMU-9 and SBL-NMU-17 peptides were characterized by a short half-life, and both yielded the 7-amino acid long metabolite (Table S1, Additional File 1). In light of Hashimoto’s study, the activities of these peptides are suggested to be maintained [39]. For NMU-9, the peptide devoid of the Gly-Tyr segment was quickly observed, while for SBL-NMU-17, only Tyr was lost. The Tic residue-containing analogue is still expected to maintain good NMUR2 agonist activity, but the NMU-9 fragment Phe-Leu-Phe-Arg-Pro-Arg-Asn-NH_2_ is not expected to do so [12, 17]. To show NMU-9 activity in non-cancer cells, namely HEK293 R2_HA cells, we tested calcium mobilisation and ERK1/2 signalling activation (Fig. S1 B-D, Additional File 2). The problem of the proteolytic stability of native NMU peptides in the cancer cell environment requires further investigation.

In the case of *NMUR1*-positive HCT116 cells, we were unable to detect significant activation of calcium mobilisation with the method used. The signals induced by NMU-9 or NMUR1 agonist were very weak (Fig. 5A; representative movies Additional File 1). The observed effect may be the result of low *NMUR1* expression (Fig. 4A) or high NMU secretion by HCT116 cells (Fig. 3A), which preferentially occupied the receptor and impaired the response of these cells after exogenous addition of NMU or receptor agonist. There is a number of mechanisms that limit GPCR signalling; one of them is receptor desensitization leading to a decrease in the response to repeated or continuous stimulation [40]. The role and function of NMU in *NMUR1*-positive HCT116 cells remain unknown but will be further explored.

In conclusion, we show for the first time that NMUR2 activation significantly induces calcium mobilisation in *NMUR2*-positive HT29 cells. Similar to endometrial and breast cancer studies [29, 30, 41–43], our data point to NMUR2 as an important functional receptor in CRC cells. TCGA data analysis showed that high *NMUR2* levels are characteristic of cancers with low differentiation and increased invasiveness, as represented by decreased *CDH1* expression and increased *MMP1* expression. Poorly differentiated CRC shows high proliferation and metastasis capacities, which have a serious impact on patient survival and prognosis [44].

Our CRC cell clone studies showed that regardless of the parental cell phenotype, NMU overexpression in cells expressing *NMUR2* enhances the metastatic potential of the cells by increasing their mobility and invasiveness via ERK1/2 kinase activation, as previously shown for other cancers [30, 32, 41, 42]. At the tissue level, mRNA data from patient samples showed significantly more events of perineural invasion in the group with both high *NMU* and *NMUR2* expression. Perineural invasion together with other types of invasion is a known mechanism of solid tumour dissemination [45] and refers to the invasion of the perineurium of large nerves by cancer cells, which is a prognostic factor of poor outcome in CRC [46].

Elevated NMU upregulates the expression of integrin subunits that are known to be drivers and supporters of the formation of metastases [47]. The observed correlation between the invasive potential of NMU-overexpressing clones and upregulation of integrin expression is consistent with the reported involvement of α2 [48], α5 [49], α6β4 [50], αV [51], β1 [52], β6 [53] and αVβ5 [54] integrins in subsequent steps leading to metastasis.

Our presented patient sample and molecular data suggest that *NMU*/*NMUR2* expression is a pathological factor associated with CRC cell invasiveness. Our previous report linked NMU to the transcription factor Snail, an inducer of early stages of metastatic transition [5], and here, we observed a significant increase in the *NMUR2* levels at early CRC stages. Thus, we can speculate that NMU/NMUR2 signal transduction is important at the early stages of the cellular mechanisms leading to CRC progression. Currently, node-negative CRC patients (stages I and II), who have overall better prognosis, do not receive adjuvant chemotherapy. However, clinical reports have indicated that a subset of these patients die of recurrence or metastatic CRC. *NMU*/*NMUR2* expression levels could be useful for more accurate clinical staging and identifying tumours with invasive potential. Consequently, assessing these expression levels could help in making decisions about who could potentially benefit from adjuvant therapy.

## Conclusions

Finally, our results show that CRC cells secrete NMU and that some of these cells have the ability to respond to NMU through NMUR2. This interaction shifts the cell phenotype towards more invasive phenotypes. CRC cells with NMUR expression from the tested panel belong mainly to the CMS3 and CMS4 cancer subtypes, both of which have poor prognosis and are difficult to treat with therapeutic intervention [3]. Further studies are needed to determine whether NMU and its receptors could be valid targets for the treatment of CRC.

## Supplementary Information

**Additional file 1: Table S1. **NMU peptides sequence and stability data (the major biodegradation sites are indicated with a backslash). **Table S2.** TaqMan Gene expression probes and primers. **Table S3. **Antibodies used for integrin staining for FACS analysis. **Supplementary methods.** Mass spectrometry analysis. Calcium mobilisation quantification. Calcium mobilisation representative movies.

**Additional file 2: Figure S1. **HEK293 R2_HA clones with exogenous* NMUR2 overexpression.* (A) *NMUR2*mRNA levels were analysed in HT29 with endogenous expression of *NMUR2* and compared to those in HEK293 R2_HA clones with exogenous expression of *NMUR2*(**p ≤ 0,01; *n* ≥ 3). (B) Ca2+ influx tested in HEK293 R2_HA cells loaded with fluo-4. Cells were treated with minimal effective concentrations of peptide, NMU-9 (0.7 µM) or an NMUR2 (SBL-NMU-17) agonist (150 nM). The changes in fluorescence were detected and analysed as described in the Methods section. The results are shown as the mean with SD (**p ≤ 0,01; *n* = 3). (C, D) ERK1/2 kinase activation in HEK293 R2_HA cells upon (C) NMU-9 or (D) NMUR2 (SBL-NMU-17) agonist treatment analysed by immunoblotting (*n* = 1). The images show representative results. **Figure S2.** ERK1/2 kinase activation in Caco-2 cells upon various (A) concentrations of NMU (*n* = 1) and (B) different incubation times (*n* = 1), analysed by immunoblotting. The images show representative results. **Figure S3. ***NMU* presence in cell lysates analysed by immunoblotting. Images show representative results. The bands were quantified by densitometry. The intensity of the NMU band was normalized to the respective GAPDH band (**p ≤ 0.01; *n* = 4). The results are shown as the medians with min-to-max ranges

**Additional file 3. **(Macro code)

**Additional file 4. **TCGA data containing patients clinical information and gene expression data.

**Additional file 5. **Paired patients data cancer / normal adjacent tissues.

**Additional file 6. **Cell lines authentication certificate.

## Data Availability

All data generated or analysed during this study are included in this published article and its supplementary information files. Representative movies are stored in the RepOD repository: 10.18150/XXYTZD

## Abbreviations

CRC: Colorectal cancer
NMU: Neuromedin U
NMUR: Neuromedin U receptor
TCGA: The Cancer Genome Atlas
GPCR: G protein-coupled receptor
EVs: Extracellular vesicles
CMS: Consensus molecular subtype
