# Supplementary material for: Neuromedin U induces an invasive phenotype in CRC cells expressing the NMUR2 receptor
Source: J Exp Clin Cancer Res. 2021 Sep 7;40:283. doi: 10.1186/s13046-021-02073-8 (PMC8422652; doi:10.1186/s13046-021-02073-8)
Supplement: Supplementary file 1 — Additional file 1: Table S1. NMU peptides sequence and stability data (the major biodegradation sites are indicated with a backslash). Table S2. TaqMan Gene expression probes and primers. Table S3. Antibodies used for integrin staining for FACS analysis. Supplementary methods. Mass spectrometry analysis. Calcium mobilisation quantification. Calcium mobilisation representative movies. [file 13046_2021_2073_MOESM1_ESM.docx]

**Table S1.** NMU peptides sequence and stability data (the major biodegradation sites are indicated with a backslash)

| **Name** | **Sequence** | **Half-life in human plasma ( t_1/2_)** |
| --- | --- | --- |
| **NMU-9** | Gly-Tyr- \ Phe-Leu-Phe-Arg-Pro-Arg-Asn-NH_2_ | 4.60 ± 0.4 min |
| **SBL-NMU-21**  NMUR1 agonist * | Ac-Tyr-Phe- \ Leu-Dmt- \ Arg-Pro-Arg-Asn-NH_2_ | 253.5 ± 6.0 min* |
| **SBL-NMU-17** NMUR2 agonist * | H-Tyr- \ Phe-Leu-Phe-Arg-Tic-Arg-Asn-NH_2_ | 3.37 ± 0.5 min |

***** De Prins A, Martin C, Van Wanseele Y, Skov LJ, Tomboly C, Tourwe D, et al. Development of potent and proteolytically stable human neuromedin U receptor agonists. Eur J Med Chem. 2018;144:887-97

**Table S2.** TaqMan Gene expression probes and primers

| **Gene name** | **TaqMan probes** | **Forward primer** | **Revers primer** |
| --- | --- | --- | --- |
| NMU | Hs00183624_m1 | - | - |
| NMUR1 | Hs00173804_m1 | 5’ GCCGGAGACAAGTGACCAAGA 3’ | 5’ TGACACGACGCTCCACATG 3’ |
| NMUR2 | Hs00173930_m1 | 5’ TCAACCTCGTCCATGTGGTG 3’ | 5’ AGCGGCGAGACAGTAGGTTA 3’ |
| NTSR1 | - | 5’ CCGTCAAGGTCGTCATACAG 3’ | 5’ GATGGTGGAGCTGACGTAGAA 3’ |
| GHSR1b | - | 5’ CTTGGGACACCAACGAGTG3’ | 5’ AGGACCCGCGAGAGAAAGC 3’ |
| GAPDH | Hs_02758991_g1 | 5’ TTGCCCTCAACGACCACTTT 3’ | 5’ TCCTCTTGTGCTCTTGCTGG 3’ |
| Actβ | Hs_01060665_g1 | 5’ CTGGAACGGTGAAGGTGACA 3’ | 5’ AAGGGACTTCCTGTAACAATGCA 3’ |

**Table S3.** Antibodies used for integrin staining for FACS analysis.

| Antibody | Conjugate | Vendor | Catalog number # |
| --- | --- | --- | --- |
| Anty-human α2 | FITC | BD Pharmingen | 555498 |
| Anty-human α4 | FITC | BD Pharmingen | 560840 |
| Anty-human α5 | FITC | SantaCruz Biotech | sc-52668 |
| Anty-human α6 | FITC | SantaCruz Biotech | sc-19622 |
| Anty-human αV | PE | R&D Systems | FAB1219P |
| Anty-human β1 | x | Milipore | MAB1959 |
| Anty-human β4 | PE | BD Pharmingen | 555720 |
| Anty-human β6 | x | R&D Systems | MAB4155 |
| Anty-human αVβ5 | PE | R&D Systems | FAB2528P |

***Mass spectrometry analysis of the immunoprecipitation products***

The gel slice was processed by following the in-gel trypsin digestion protocol, as described in detail by Shevchenko et al. (2007)**, with the exception that chymotrypsin was used instead of trypsin. The resulting chymotryptic peptides were subsequently measured using the ion source of the Q Exactive™ Hybrid Quadrupole-Orbitrap Mass Spectrometer (Thermo Electron Corp) coupled to a nano-HPLC system fitted with a RP-18 column (Waters). The mass spectrometer was operated in a data-dependent mode with a selected mass range of 300–2000 mass/charge (m/z). The raw result files were processed directly using the PEAKS X Studio software. The fragmentation spectra were searched against a user-defined database, created by fetching the FASTA records for all isoforms of NMU protein from the ENSEMBL database. The PEAKS X Studio search was performed with standard parameters.

**Shevchenko, A.; Tomas, H.; Havlis, J.; Olsen, J.V.; Mann, M. In-gel digestion for mass spectrometric characterization of proteins and proteomes. Nat Protoc 2006, 1, 2856-2860, doi:10.1038/nprot.2006.468.

***Calcium mobilisation quantification***

Quantification of the calcium mobilisation were performed with the use of FIJI image processing package***. Images of nuclei were used to create lists of regions of interests (ROIs). Fluorescence intensity change over time was measured in each ROI in corresponding movie sequence of Fluo-4 stained cells. To acquire the change of background fluorescence over time in each movie sequence, additional ROIs were created by the analyst manually in the sites devoid of cells. Macro code for the workflow is contained in Additional File 3. The averaged intensity value of the background in each time-frame was subtracted from the intensity values obtained for the cells. Such background-corrected values of fluorescence intensity were used for further calculations. To calculate the effect of a tested compound on the calcium flux, an average intensity of fluorescence of each cell prior to addition of the compound was subtracted from a maximum value of intensity after addition of the compound. Based on the values calculated for control experiments (cells treated with medium), threshold cut-off values were defined for each cell line. Final results are presented as the percentage of cells above the threshold.

*** Schindelin, J.; Arganda-Carreras, I.; Frise, E.; Kaynig, V.; Longair, M.; Pietzsch, T.; Preibisch, S.; Rueden, C.; Saalfeld, S.; Schmid, B., et al. Fiji: an open-source platform for biological-image analysis. *Nat Methods* **2012**, *9*, 676-682, doi:10.1038/nmeth.2019

***Calcium mobilisation representative movies***

Representative movies listed below are available at the link to RepOD repository:

Przygodzka, Patrycja, 2021, "Calcium mobilisation upon NMURs activation in CRC cells", <https://doi.org/10.18150/XXYTZD>, RepOD, V1

- 01a_HCT116_NMU9_1uM – HCT116 cells treated with NMU-9 (1 µM)
- 01b_HCT116_agonist21_40nM – HCT116 cells treated with NMUR1 agonist (SBL-NMU-21; 40 nM)
- 02a_HT29_NMU9_07uM - HT29 cells treated with NMU-9 (0.7 µM)
- 02b_HT29_NMU9_1uM – HT29 cells treated with NMU-9 (1 µM)
- 03a_HT29_agonist17_150nM - treated with NMUR2 agonist (SBL-NMU-17; 150 nM)
- 03b_HT29_agonist17_200nM - treated with NMUR2 agonist (SBL-NMU-17; 200 nM)
- 04a_HEKR2_NMU9_07uM – HEK293 R2_HA clone treated with NMU-9 (0.7 µM)
- 04b_HEKR2_agonist17_150nM - HEK293 R2_HA clone treated with NMUR2 agonist (SBL-NMU-17; 150 nM)
