# Supplementary material for: Neuromedin U induces an invasive phenotype in CRC cells expressing the NMUR2 receptor
Source: J Exp Clin Cancer Res. 2021 Sep 7;40:283. doi: 10.1186/s13046-021-02073-8 (PMC8422652; doi:10.1186/s13046-021-02073-8)
Supplement: Supplementary file 2 — Additional file 2: Figure S1. HEK293 R2_HA clones with exogenous NMUR2 overexpression. (A) NMUR2mRNA levels were analysed in HT29 with endogenous expression of NMUR2 and compared to those in HEK293 R2_HA clones with exogenous expression of NMUR2(**p ≤ 0,01; n ≥ 3). (B) Ca2+ influx tested in HEK293 R2_HA cells loaded with fluo-4. Cells were treated with minimal effective concentrations of peptide, NMU-9 (0.7 µM) or an NMUR2 (SBL-NMU-17) agonist (150 nM). The changes in fluorescence were detected and analysed as described in the Methods section. The results are shown as the mean with SD (**p ≤ 0,01; n = 3). (C, D) ERK1/2 kinase activation in HEK293 R2_HA cells upon (C) NMU-9 or (D) NMUR2 (SBL-NMU-17) agonist treatment analysed by immunoblotting (n = 1). The images show representative results. Figure S2. ERK1/2 kinase activation in Caco-2 cells upon various (A) concentrations of NMU (n = 1) and (B) different incubation times (n = 1), analysed by immunoblotting. The images show representative results. Figure S3. NMU presence in cell lysates analysed by immunoblotting. Images show representative results. The bands were quantified by densitometry. The intensity of the NMU band was normalized to the respective GAPDH band (**p ≤ 0.01; n = 4). The results are shown as the medians with min-to-max ranges [file 13046_2021_2073_MOESM2_ESM.docx]

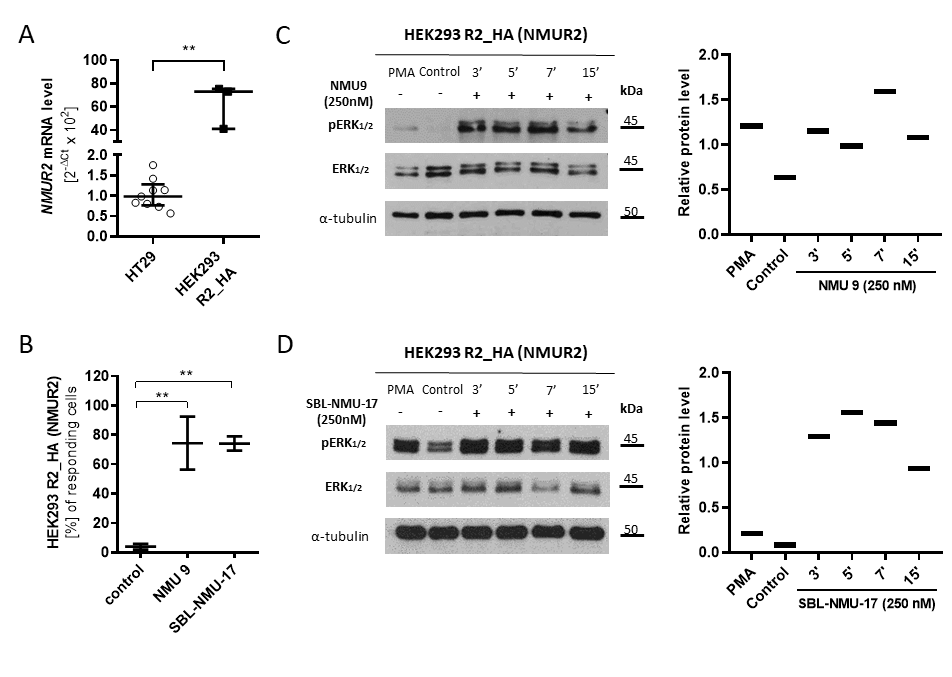


**Fig. S1 *HEK293 R2_HA* *clones with exogenous NMUR2 overexpression.*** (A) *NMUR2* mRNA levels were analysed in HT29 with endogenous expression of *NMUR2* and compared to those in HEK293 R2_HA clones with exogenous expression of *NMUR2* (**p ≤ 0,01; n ≥ 3). (B) Ca2+ influx tested in HEK293 R2_HA cells loaded with fluo-4. Cells were treated with minimal effective concentrations of peptide, NMU-9 (0.7 µM) or an NMUR2 (SBL-NMU-17) agonist (150 nM). The changes in fluorescence were detected and analysed as described in the Methods section. The results are shown as the mean with SD (**p ≤ 0,01; n = 3). (C, D) ERK1/2 kinase activation in HEK293 R2_HA cells upon (C) NMU-9 or (D) NMUR2 (SBL-NMU-17) agonist treatment analysed by immunoblotting (n = 1). The images show representative results.


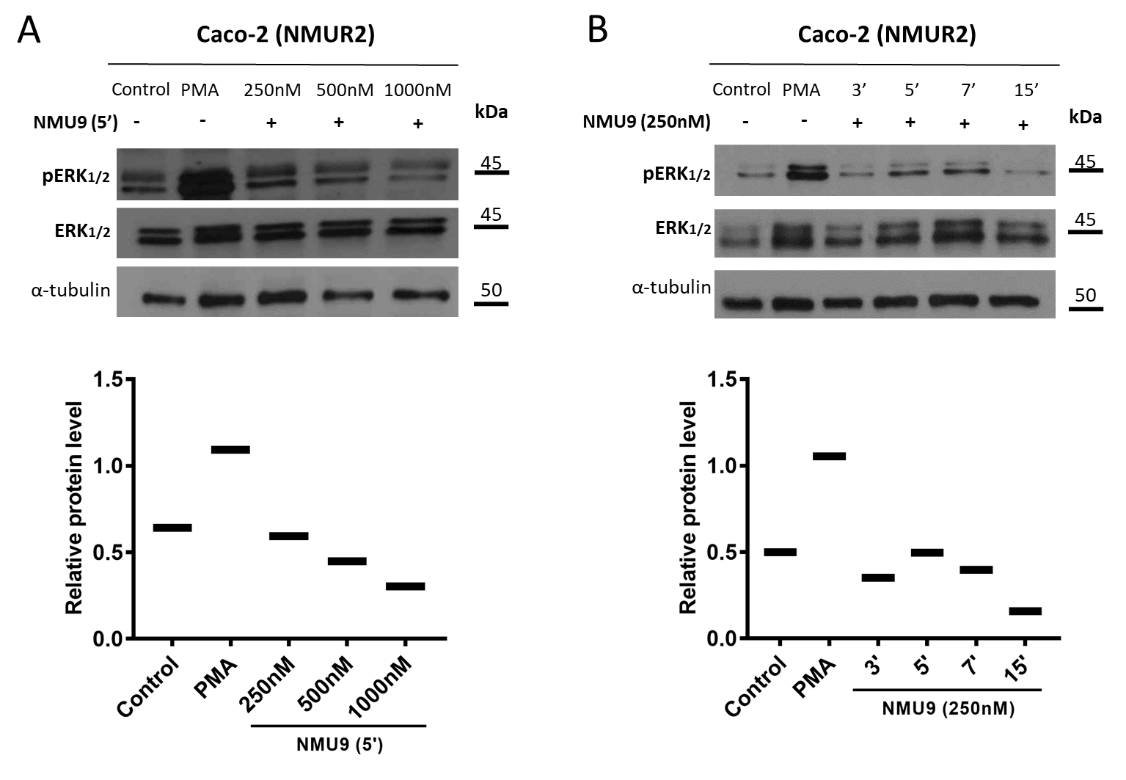


**Fig. S2** ERK1/2 kinase activation in Caco-2 cells upon various (A) concentrations of NMU (n = 1) and (B) different incubation times (n = 1), analysed by immunoblotting. The images show representative results.


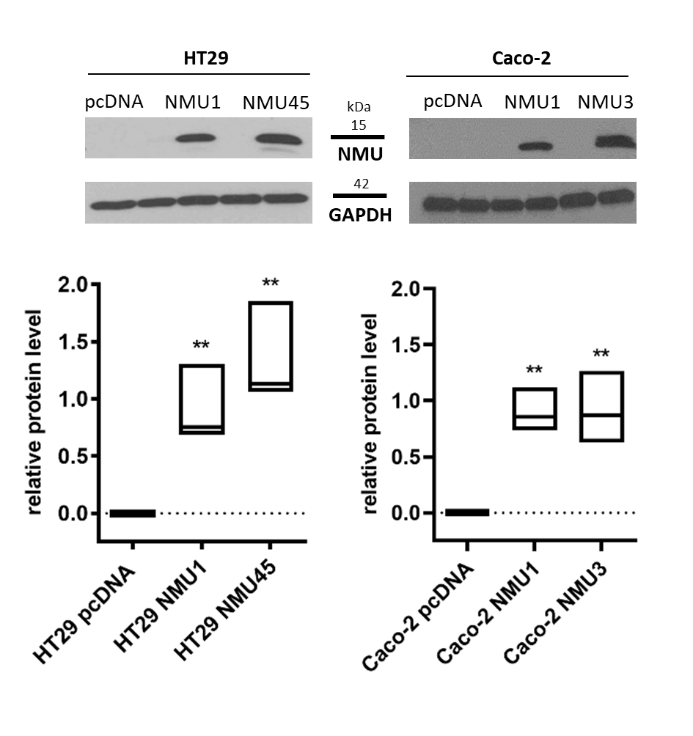


**Fig. S3.** ***NMU presence in cell lysates analysed by immunoblotting.*** Images show representative results. The bands were quantified by densitometry. The intensity of the NMU band was normalized to the respective GAPDH band (**p ≤ 0.01; n = 4). The results are shown as the medians with min-to-max ranges.
