# Supplementary material for: Neuromedin U induces an invasive phenotype in CRC cells expressing the NMUR2 receptor
Source: J Exp Clin Cancer Res. 2021 Sep 7;40:283. doi: 10.1186/s13046-021-02073-8 (PMC8422652; doi:10.1186/s13046-021-02073-8)
Supplement: Supplementary file 6 — Additional file 6. Cell lines authentication certificate. [file 13046_2021_2073_MOESM6_ESM.pdf]

Eurofins Genomics

Eurofins Genomics Europe Applied Genomics GmbH, Anzinger Str. 7 a, D-85560 Ebersberg

Dr. Patrycja Przygodzka
Institute of Medical Biology of PAS, Cellular Signaling
ulica Lodowa 106
93-232, Lodz
Poland

Certificate
Cell Line Authentication Test
Order ID: 11107394206

Report date: 12.05.2021

Method:
DNA isolation was carried out from cell pellet (cell layer).
Genetic characteristics were determined by PCR-single-locus-technology.
16 independent PCR-systems D8S1179, D21S11, D7S820, CSF1PO, D3S1358, TH01, D13S317, D16S539, D2S1338, AMEL, D5S818, FGA, D19S433, vWA, TPOX and D18S51 were investigated.
(ASN-0002 core markers are colored grey, Thermo Fisher, AmpFlSTR® Identifiler® Plus PCR Amplification Kit)
In parallel, positive and negative controls were carried out yielding correct results.

Result:

**Client Sample Name**	HT29_IBM	HCT15_IBM	HCT116_IBM	Caco-2_IBM	SW480_IBM	SW620_IBM
**Sample Code**	21_ZE_000615	21_ZE_000616	21_ZE_000617	21_ZE_000618	21_ZE_000619	21_ZE_000620
D8S1179	10,16	15,15	11,12,14,15	12,14	13,13	13,13
D21S11	29,30	29,32.2	29,30	30,32	30,30.2	30,30.2
D7S820	10,10	10,12	11,12	11,12	8,8	8,9
CSF1PO	11,12	12,12	7,10,11	11,11	13,14	13,14
D3S1358	15,17	17,17	11,12,17,18,19	14,17	15,15	16,16
TH01	6,9	7,9.3	8,9	6,6	8,8	8,8
D13S317	11,12	8,11	10,11,12,13	11,13,14	12,12	12,12
D16S539	11,12	12,13	11,12,13,14	12,13	13,13	9,13
D2S1338	19,23	17,25	16,16	17,25	17,24	17,24
D19S433	14,14	14,16	12,13	15,15	13,13	13,13
vWA	17,19	18,19	17,21,22,23	16,18	16,16	16,16
TPOX	8,9	8,11	8,9	9,11	11,11	11,11
D18S51	13,13	11,17	16,17,18	12,12	13,13	13,13
AMEL	X,X	X,Y	X,Y	X,X	X,X	X,X
D5S818	11,12	13,13	10,11,12	12,13	13,13	13,13
FGA	20,22	22,22	17,18,22,23,24	19,19	24,24	24,24
**Database Name**	HT-29	HCT-15	HCT 116*	CACO-2	SW-480	SW620

The table shows the result of the cell line analysis and the comparison with the online database of the DSMZ (http://www.dsmz.de/de/service/services-human-and-animal-cell) and the Cellosaurus database (https://web.expasy.org/cellosaurus). Please note that only the PCR-systems according to ANSI/ATCC standard ASN-0002 were aligned (D5S818, D13S317, D7S820, D16S539, VWA, TH01, TPOX, CSF1PO, AMEL - colored grey).

*The sample could be identified as the stated cell line, although one or several PCR-Systems did show additional signals (see table). These signals could occur due to contamination of the cell line or possible mutations.

This report was created automatically and is therefore valid without a signature.
